# Complex DNA repair pathways as possible therapeutic targets to overcome temozolomide resistance in glioblastoma

**DOI:** 10.3389/fonc.2012.00186

**Published:** 2012-12-05

**Authors:** Koji Yoshimoto, Masahiro Mizoguchi, Nobuhiro Hata, Hideki Murata, Ryusuke Hatae, Toshiyuki Amano, Akira Nakamizo, Tomio Sasaki

**Affiliations:** Department of Neurosurgery, Graduate School of Medical Sciences, Kyushu UniversityFukuoka, Japan

**Keywords:** TMZ, DNA repair, PARP, homologous recombination, chemoresistance

## Abstract

Many conventional chemotherapeutic drugs exert their cytotoxic function by inducing DNA damage in the tumor cell. Therefore, a cell-inherent DNA repair pathway, which reverses the DNA-damaging effect of the cytotoxic drugs, can mediate therapeutic resistance to chemotherapy. The monofunctional DNA-alkylating agent temozolomide (TMZ) is a commonly used chemotherapeutic drug and the gold standard treatment for glioblastoma (GBM). Although the activity of DNA repair protein O6-methylguanine-DNA methyltransferase (MGMT) has been described as the main modulator to determine the sensitivity of GBM to TMZ, a subset of GBM does not respond despite MGMT inactivation, suggesting that another DNA repair mechanism may also modulate the tolerance to TMZ. Considerable interest has focused on MGMT, mismatch repair (MMR), and the base excision repair (BER) pathway in the mechanism of mediating TMZ resistance, but emerging roles for the DNA strand-break repair pathway have been demonstrated. In the first part of this review article, we briefly review the significant role of MGMT, MMR, and the BER pathway in the tolerance to TMZ; in the last part, we review the recent publications that demonstrate possible roles of DNA strand-break repair pathways, such as single-strand break repair and double-strand break repair, as well as the Fanconi anemia pathway in the repair process after alkylating agent-based therapy. It is possible that all of these repair pathways have a potential to modulate the sensitivity to TMZ and aid in overcoming the therapeutic resistance in the clinic.

## INTRODUCTION

Glioblastoma (GBM) is the most common malignant tumor of the brain in adults (Wen and Kesari, 2008). Due to the invasive nature of the tumor, complete surgical extirpation of the tumor cell is rarely achieved. The current gold standard for treatment of patients with GBM is multidisciplinary management by maximum tumor resection with concomitant temozolomide (TMZ) chemotherapy and radiotherapy (Reardon et al., 2006; Stupp et al., 2006). Although TMZ chemotherapy significantly prolongs the survival of patient with newly diagnosed GBM, the median survival is still between 12 and 15 months (Reardon et al., 2006; Stupp et al., 2009), indicating that overcoming TMZ resistance is a critical issue to improve treatment outcomes. Chemoresistance in the tumor can be caused by several cellular factors such as increased efflux ability of chemotherapeutic drugs, expression of anti-apoptotic proteins, and activation of DNA repair pathways.

Although all of the conventional chemotherapeutic drugs, including TMZ, exert their cytotoxic function by damaging DNA and inducing cell death in tumors, normal cells possess several DNA repair systems to combat DNA damage (Friedman et al., 2000; Fu et al., 2012). In cancer cells, DNA repair systems are frequently altered compared to normal cells. Some DNA repair pathways are impaired due to genomic mutations and epigenetic events and therefore depend on other repair pathways to recover from the DNA damage. The DNA repair activity can have a negative effect on the sensitivity to cytotoxic chemotherapeutic drugs (Madhusudan and Hickson, 2005; Ding et al., 2006; Helleday et al., 2008). Cytotoxicity of TMZ is provoked by TMZ-generated O6-methylguanine-DNA adducts, which is repaired by the O6-methylguanine-DNA methyltransferase (MGMT) repair enzyme, thereby suppressing cytotoxicity. Thus, these data suggest that activation of the MGMT enzyme is associated with resistance to TMZ therapy (Friedman et al., 2000; Sarkaria et al., 2008; Fu et al., 2012). Indeed, it has been reported that GBM patients with MGMT promoter methylation respond better than unmethylated patients for the concomitant TMZ and radiotherapy, making MGMT methylation a critical biomarker for predicting sensitivity to TMZ therapy. However, emerging evidence indicates that MGMT-independent DNA repair pathways play a role in mediating the therapeutic response to TMZ in GBM (Kondo et al., 2010b; Alexander et al., 2012; Bartek et al., 2012).

## DNA REPAIR PATHWAYS AS A TARGET OF CANCER THERAPY

Cellular DNA is exposed to various kinds of DNA damage inducers. Reactive oxygen species (ROS), ultraviolet (UV) light, and many kinds of chemicals, for example, carcinogens from cigarette smoking, are well known inducers of DNA damage *in vivo*. To combat this DNA damage, normal cells have intrinsic DNA damage response (DDR) systems to protect them from endogenous and exogenous DNA damage inducers (Harper and Elledge, 2007; Jackson and Bartek, 2009; Ciccia and Elledge, 2010; Lord and Ashworth, 2012). DDR systems provoke different biological processes depending on the type and strength of the damage, such as DNA repair, cell cycle arrest, cell death, and cell senescence. The cell cycle is arrested during the DNA repair process as long as normal DNA repair capacity is preserved. However, when the normal repair process is impaired or altered, DNA damage is not repaired, triggering cell death in the tumor cells. Cytotoxic anti-cancer drugs are used in the clinic to induce DNA damage in cells for the purpose of triggering cell death directly and indirectly following DNA replication. Therefore, the DNA repair status in the tumor cells is associated with the therapeutic response to the anti-cancer drug, establishing DNA repair pathways as promising targets for cancer treatment, including GBM (Madhusudan and Hickson, 2005; Ding et al., 2006; Helleday et al., 2008; Al-Ejeh et al., 2010; Kondo et al., 2010b; Alexander et al., 2012; Aziz et al., 2012; Bouwman and Jonkers, 2012).

DNA damage is repaired through a variety of DNA repair pathways, depending on the type of DNA damage. DNA repair pathways consist of the direct repair (DR), base excision repair (BER), nucleotide repair (NER), mismatch repair (MMR), and DNA strand-break repair pathways, among others (Harper and Elledge, 2007; Jackson and Bartek, 2009; Ciccia and Elledge, 2010). The Fanconi anemia (FA) pathway repairs interstrand cross-links (ICLs). DNA repair pathway functions are redundant in the context of cellular DNA damage, and have back-up systems, making the response to DNA damage more complex. In addition, it should be stressed that even though chemotherapeutic drugs, including TMZ, do not directly induce DNA double-strand breaks (DSBs), unrepaired damaged bases and nucleotides ultimately cause DSBs. Thus, the DNA strand-break repair pathway should also be considered for investigation into the mechanisms underlying resistance to chemotherapy. Another key concept for targeting the DNA repair strategy is the synthetic lethal approach (Moeller et al., 2009; Reinhardt et al., 2009; Chan and Giaccia, 2011). As mentioned earlier, it is possible that if one of the critical DNA repair pathways is impaired, another pathway in the tumor cell will be activated. In these situations, only attenuating the affected DNA repair pathway effectively confers a strong cytotoxic effect that prevents tumor cells from replicating, but does not affect normal cells. This treatment strategy, called “synthetic lethality,” is effective as a targeted therapy for a DNA repair pathway (Reinhardt et al., 2009). Indeed, there have been numerous reports demonstrating the clinical utility of the concept of the synthetic lethality approach in various solid tumors (Chan and Giaccia, 2011).

## DEFINITE ROLES OF MGMT, MMR, AND BER PATHWAYS IN THE TOLERANCE OF TMZ TREATMENT

**FIGURE 1:**
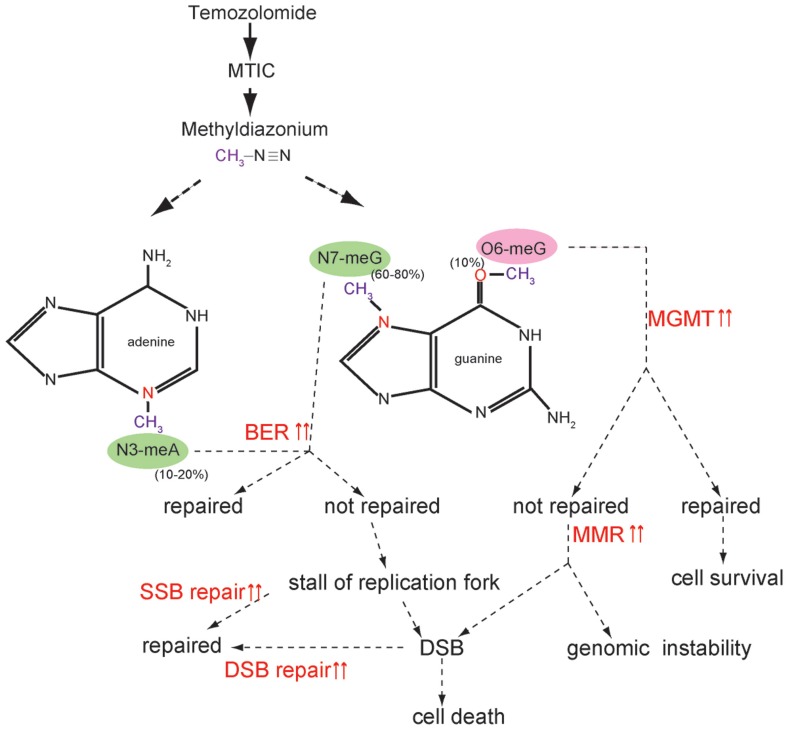
**Temozolomide function and activated DNA repair pathway**. Temozolomide (TMZ) is chemically converted to MTIC (5-3-(methyltriazen-1-yl) imidazole-4-carboximide) at physiologic pH and degrades to a methyldiazonium cation, which transfers a methyl group to DNA. The most common site of methylation is N7-MeG (60–80%) followed by N3-MeA (10–20%) and O6-MeG (5–10%). When active MGMT is present, O6-MeG is repaired without cytotoxicity. When MGMT is inactivated or does not have the potential to completely repair O6-MeG, unrepaired O6-MeG is continuously repaired by the futile cycle of MMR, which ultimately induces cell death by provoking double-strand breaks (DSB). When MMR does not function properly, genomic instability is amplified. N7-MeG and N3-MeA is repaired by BER. If not repaired, alkylated bases cause a replication stall and collapse of the replication fork, generating single-strand breaks (SSBs), which ultimately induce DSB. It is possible that SSB and DSB repair pathways are activated and diminish the cytotoxic effects of TMZ.

Temozolomide is a monofunctional SN-1 alkylating agent that can modify nitrogen atoms in the DNA ring and the extracyclic oxygen group. TMZ is chemically converted to MTIC (5-3-(methyltriazen-1-yl) imidazole-4-carboximide) at physiologic pH and degrades to methyldiazonium cation, which transfers methyl groups to DNA (Friedman et al., 2000). The common site of methylation is at the N7 position of guanine (N7-MeG; 60–80%) followed by the N3 position of adenine (N3-MeA; 10–20%) and the O6 position of guanine (O6-MeG; 5–10%; Friedman et al., 2000; Fu et al., 2012; **Figure 1**).

### O6-METHYLGUANINE-DNA METHYLTRANSFERASE

Although O6-MeG is the least frequently alkylated DNA adduct, O6-MeG is a major cytotoxic adduct induced by TMZ treatment (**Figure 1**). Therefore, the DR pathway by MGMT, which removes TMZ-generated O6-MeG by transferring the methyl adducts to its own cysteine residues, can mediate tolerance to TMZ treatment. This theoretical function has been validated by many investigators using a preclinical model (Pegg, 1990; Gerson, 2004), and MGMT methylation has been linked to therapeutic sensitivity to TMZ in the clinical study (Hegi et al., 2005; Stupp et al., 2009). Given that MGMT inactivation is a molecular biomarker of good response to TMZ, several strategies have been investigated to reduce the MGMT activity for the purpose of enhancing TMZ sensitivity (Hegi et al., 2008). O^6^-benzylguanine (O^6^-BG) has been known to inhibit MGMT activity and enhance the cytotoxicity of TMZ *in vitro* (Wedge and Newlands, 1996). However, the result of recent clinical trial demonstrated that the combination of O^6^-BG with TMZ did not show clinical benefit for recurrent and TMZ-resistant malignant glioma patients (Quinn et al., 2009). In addition, because alternative TMZ dosing regimens reduce MGMT activity, several dose-dense (dd) TMZ regimens have been investigated in the clinic (Wick et al., 2009; Neyns et al., 2010). Although the clinical significance of these dd TMZ regimens has not been determined, the recent study did not demonstrate the improved efficacy of dd TMZ regimens (Gilbert et al., 2011). Further studies are underway to evaluate the significance of dd TMZ regimens.

### MISMATCH REPAIR

**FIGURE 2:**
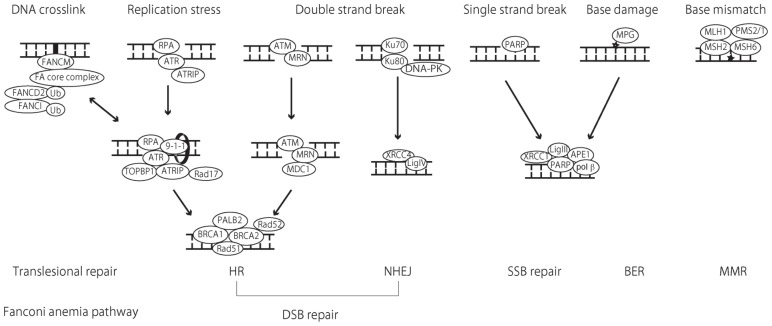
**Schematic illustration of DNA repair pathways and implicated proteins**. Mismatch repair (MMR) functions in the repair of base substitution mutations as well as small insertions/deletions caused by replication errors. The heterodimeric complex of MSH2 and MSH6 recognizes base mismatches and single-base insertions/deletions, whereas the MSH2 and MSH6 complex (not described in this schema) detect larger insertions/deletions. These complexes recruit another heterodimeric complex made up of MLH1 and PMS2 to initiate the repair process by excision of mismatches and insertions/deletions and re-synthesis of the DNA strand. In base excision repair (BER), a damaged base is recognized by a DNA glycosylase enzyme. Of all DNA glycosylase, DNA glycosylase (MPG) [alkylpurine-DNA-*N*-glycosylase (APNG)] recognizes and removes alkylated bases before apurinic/apyrimidinic endonuclease 1 (APE1) recognizes the abasic sites and cleaves the 5′ end of the DNA. In the major BER pathway, as a short-patch pathway, DNA polymerase β (polyβ) excises the 3′ end of the DNA and fills the gap. Finally, the XRCC1-ligase III complex catalyzes the formation of phosphodiester bonds and completes the repair process. Poly (ADP-ribose) polymerase (PARP) recognizes single-strand breaks and initiates the repair process in an overlapping manner with BER. For DSB repair, non-homologous end-joining (NHEJ) and homologous recombination (HR) are utilized in the repair mechanism. In NHEJ, DSB are recognized by the Ku70/80 proteins, which bind and activate the protein kinase DNA-PKcs, recruiting XRCC4 and DNA ligase IV (Lig IV) to seal the gap. In HR, the MRE-11-RAD50-NBS1 (MRN) complex act as a sensor of DSB and binds to the break ends. Ataxia telangiectasia mutated (ATM) is then recruited to the sites of the breaks where ATM is phosphorylated and activates H2AX, generating phosphorylated H2AX (γ H2AX). DNA damage checkpoint protein 1 (MDC1) binds γ H2AX and the recombination process begins. Replication stress causes single-stranded DNA and induces subsequent DSB. In this case, single-stranded DNA is bound by replication protein A (RPA), which recruits ATR and ATR-interacting protein (ATRIP) to the break site. Rad17 and 9-1-1 are subsequently recruited to ATR-ATRIP, which is finally activated with the aid of DNA topoisomerase II-binding protein 1 (TOPBP1). In the process of HR, the BRCA1 complex with MDC1 has an essential role in damage detection. The subsequent BRCA1-BRCA1-partner and localizer of BRCA2 (PALB2)-BRCA2 complex is important in mediating HR. BRCA2-mediated Rad51 recruitment is crucial for HR. Rad52 is also needed for RAd51 to enable access to single-stranded DNA coated with RPA. The Fanconi anemia (FA) pathway functions during translesion DNA synthesis (TLS) in the repair of DNA interstrand cross-links (ICLs). ICLs are recognized by FANCM and the FA core complex, which monoubiquitinates FANCD2 and FANCI, thereby recruiting nucleases and other mediator proteins to facilitate the repair process. Recent studies have shown that the FA pathway regulates not only ICL, but also HR, possibly mediating BRCA2 and Rad51 function. The MGMT and nucleotide excision repair (NER) pathways are not described in this schema.

Mismatched repair mends DNA damage and base mismatches as well as incorrect insertions/deletions arising from DNA replication. In MMR, base mismatches are recognized by the heterodimers of MSH2 and MSH6, which recruit another heterodimeric complex of MLH1 and PMS2, thereby regulating the repair process (Stojic et al., 2004a; **Figure 2**). TMZ-induced O6-MeG is not repaired by MGMT. Unrepaired O6-MeG can pair with cytosine (C) or thymidine (T). The O6-MeG/C or O6-MeG/T is recognized by the MMR system and only newly synthesized strands are excised, keeping O6-MeG intact. When another strand is generated, this repair cycle is repeated. This “futile cycle” provokes replication fork arrest during DNA synthesis, and cytotoxicity is induced by causing DSB formation (Wang and Edelmann, 2006; Fu et al., 2012; **Figure 1**). Thus, the normal function of the MMR pathway is a prerequisite for O6-MeG-induced cytotoxicity. The inactivation of MMR has been associated with tolerance to the cytotoxic effects of alkylating agents (Friedman et al., 1997; D’Atri et al., 1998; Fink et al., 1998; Kinsella, 2009).

Although only a few gene mutations of MMR genes such as MSH2, MSH6, MLH1, and PMS2 in GBM have been discovered, recent investigations have revealed that MSH6 mutations arise in GBMs during TMZ therapy and mediate TMZ resistance. Hunter et al. (2006) identified somatic MSH6 mutations in recurrent GBM tissues after alkylating therapy with no mutations in matched pretreatment samples, suggesting that MSH6 mutations mediate clinical resistance to TMZ. Cahill et al. (2007) also showed that although MSH6 mutations were not observed in any pretreatment GBM specimens, 3 of 14 (21%) recurrent cases had somatic mutations, and 7 of 17 (41%) recurrent tumors showed decreased expression of MSH6, compared with the matched pretreatment specimens, after concomitant TMZ and radiotherapy, indicating that loss of MSH6 function is associated with tumor recurrence during TMZ treatment. Their subsequent studies confirmed MSH6 mutations in post-treatment TCGA samples, but the absence of mutations in matched pretreatment samples, and demonstrated that MSH6 mutations mediate TMZ resistance in an *in vitro* tumor model (Yip et al., 2009). Moreover, the German Glioma Group recently reported that reduced expression of MMR protein was associated with recurrence of GBM after TMZ treatment (Felsberg et al., 2011). These results support the notion that a normal MMR system is indispensable for TMZ-induced cytotoxicity.

### BASE EXCISION REPAIR

Base excision repair mends DNA from the damaging effects of oxidation, alkylation, deamination, and single-strand breaks (SSBs). TMZ-induced N7-MeG and N3-MeA is sensed by the BER pathway and the repair process is initiated (Fu et al., 2012; **Figure 1**). BER has been reported to play a role in the tolerance of TMZ resistance (Trivedi et al., 2005). DNA glycosylases play a role in recognizing and excising damaged bases, and initiating the repair process. Among the 11 mammalian DNA glycosylases, *N*-methylpurine DNA glycosylase (MPG) [or alkylpurine-DNA-*N*-glycosylase (APNG)] is a DNA glycosylase that excises alkylated bases from DNA (Jacobs and Schar, 2012). After damaged bases are removed by DNA glycosylase, apurinic/apyrimidinic endonuclease 1 (APE1) recognizes the abasic sites and cleaves the 5′ end of the DNA. Then, DNA polymerase β (poly β) along with the XRCC1-Ligase III (Lig III) complex complete the repair process (Fortini and Dogliotti, 2007; Svilar et al., 2011; **Figure 2**).

The expression of DNA glycosylases has been associated with the sensitivity to alkylating agents. Agnihotri et al. (2012) recently demonstrated that APNG expression modulates the repair of TMZ-induced DNA damage and APNG confers resistance to TMZ in *in vitro* and *in vivo* models of GBM. They also demonstrated that APNG is epigenetically silenced in the GBM tissues and APNG confers poor overall survival in GBM patients, indicating that APNG can be a molecular target for overcoming TMZ resistance. It has also been shown that inhibition of polyβ increases the sensitivity to TMZ-induced toxicity, indicating that the BER pathway may be a therapeutic target for enhanced TMZ sensitivity (Trivedi et al., 2005).

## POSSIBLE ROLES OF OTHER REPAIR PATHWAYS IN MEDIATING TMZ RESISTANCE

### SINGLE-STRAND BREAK REPAIR PATHWAY

Poly(ADP-ribose) polymerase (PARP) recognizes SSBs and initiates the repair process. PARP uses NAD+ as a substrate to catalyze the covalent attachment of PAR polymers on itself and other acceptor proteins, recruiting DNA repair proteins to the damaged site, which facilitates downstream repair processes (Kim et al., 2005; Schreiber et al., 2006; De Vos et al., 2012; Luo and Kraus, 2012). After SSBs are recognized by PARP, the repair process can be initiated by a shared process with the BER pathway (Almeida and Sobol, 2007; **Figure 2**). Thus, PARP is a crucial regulator of not only SSB repair, but also of the BER pathway, which makes PARP a therapeutic target in modulating the DNA repair system. Targeted drugs that inhibit PARP activity have been developed, and it has been shown that these PARP inhibitors significantly enhance the cytotoxicity of conventional anticancer drugs including those used to treat GBM (Malanga and Althaus, 2005; Chalmers, 2010; Heitz et al., 2010; Leonetti et al., 2012). Due to these therapeutic effects in preclinical studies, several trials are underway using PARP inhibitors for GBM patients (Leonetti et al., 2012).

The rationale for this treatment is to potentiate the therapeutic effects of DNA-damaging agents using PARP inhibitors. It has been shown that a combination of TMZ and PARP inhibitors enhance the cytotoxic effects of TMZ in GBM preclinical models (Tentori et al., 2002, 2003; Miknyoczki et al., 2003; Calabrese, 2004). In addition, it has been shown that PARP inhibition can overcome the resistance to TMZ in MMR-deficient cells (Curtin et al., 2004; Cheng et al., 2005). Based on these promising results, several clinical trials of PARP inhibitors for GBM are ongoing to increase the therapeutic effect of TMZ (Chalmers, 2010; Leonetti et al., 2012).

Another promising strategy for PARP inhibitor therapy is monotherapy under the concept of synthetic lethality (Mangerich and Burkle, 2011). A growing body of evidence suggests that PARP inhibition has a significant cytotoxic effect for the BRCA1- and BRCA2-mutated breast and ovarian cancers (Farmer et al., 2005; Fong et al., 2009; Venkitaraman, 2009; Rehman et al., 2010). In BRCA-mutated tumors, the DSB repair pathway by homologous recombination (HR) processes (described in the next section) is impaired. Therefore, the DNA repair process is dependent on the PARP repair pathway, where PARP inhibition has detrimental effects on the tumor cells. Although this synthetic lethal approach seems to be a promising therapy, BRCA mutation is rarely reported in GBM (Parsons et al., 2008; TCGA, 2008). However, it has recently been reported that mutation of tumor suppressor PTEN is linked to impairment of a HR repair pathway, implicating synthetic lethal targeting of PTEN mutant cells with PARP inhibitors (Shen et al., 2007; Mendes-Pereira et al., 2009; McEllin et al., 2010; Ming and He, 2012). Given that PTEN mutation is identified in about one-third of GBM patients (Parsons et al., 2008; TCGA, 2008), PARP inhibitor monotherapy has the potential to be an effective treatment strategy for the PTEN-mutated GBM. Recently, several preclinical studies demonstrated that up-regulation of a HR pathway correlates with the sensitivity to combination therapy of TMZ and PARP inhibitors (Liu et al., 2009; Loser et al., 2010). Further investigation is needed to evaluate the status of HR in PTEN-mutated GBM and the response to TMZ in combination with a PARP inhibitor.

### DOUBLE-STRAND BREAK PATHWAY

Double-strand break is repaired by two major mechanisms: non-homologous end-joining (NHEJ) and HR (**Figure 2**). NHEJ repairs DNA blunt-ends breaks throughout the cell cycle and repairs DSB even when there are no templates for recombination regardless of the cell cycle. In contrast, the HR pathway repairs the DSB by homology-mediated recombination processes using sister-chromatid sequences as the template. Thus, HR functions only in the S and G2 phases of the cell cycle, indicating that HR can function only in proliferating tumor cells. With the exception of a topoisomerase II inhibitor and radiotherapy, most of the anticancer drugs, such as alkylating agents and replication inhibitors, do not induce DSB directly. However, when bases damaged by cytotoxic drugs are not repaired by BER or the single-strand repair pathway, DNA replication errors cause replication stress (replication stalls or a replication fork collapses), resulting in DSB. Accumulating evidence indicates that the HR process may play an important role in mediating DSB repair after replication stress (Helleday et al., 2007; Helleday, 2010; Roy et al., 2012). Theoretically, TMZ ultimately induces tumor cell death by provoking DSB. Taken together, it is possible that the status of DSB repair activity in the tumor cell has the potential to determine the clinical response to TMZ treatment. Indeed, there have been several reports of the significant role of DSB repair proteins in determining the sensitivity to TMZ in preclinical GBM models; these will be described below.

Within a few minutes of damage, NHEJ repair blunt DSB, which are recognized by the Ku70/80 proteins and bind with the protein kinase DNA-PKcs, recruiting XRCC4 and DNA ligase IV (Lig IV) to seal the gap (**Figure 2**). Given that NHEJ functions to blunt the DSB, independent of the tumor cell cycle, a significant role in the repair process of radiotherapy in GBM has been reported (Mukherjee et al., 2009; Zhuang et al., 2011). Although a possible role for the NHEJ pathway in the sensitivity of TMZ in GBM cells has been reported, only a few reports have documented this result (Fischer and Meese, 2007; Roos et al., 2007; Kondo et al., 2010a).

Homologous recombination is initiated by 5′–3′ resection at two-ended DSB, generating single-stranded DNA. The MRE-11-RAD50-NBS1 (MRN) complex acts as a sensor of DSB and binds to a break at the ends. Ataxia telangiectasia mutated (ATM) is then recruited to the sites of the breaks where ATM is phosphorylated and activates H2AX, generating phosphorylated H2AX (γ H2AX). DNA damage checkpoint protein 1 (MDC1) binds γ H2AX and is processed by a recombination process (**Figure 2**). In contrast to the two-ended DSB described above, DNA replication stress is known to cause one-ended DSB, generating single-stranded DNA (Helleday et al., 2007). In these situations, ATM and Rad3-related (ATR) signaling is activated to repair one-ended DSB by HR (Cimprich and Cortez, 2008; Flynn and Zou, 2011). Single-stranded DNA is bound by replication protein A (RPA), which recruits ATR and ATR-interacting protein (ATRIP) to the break site. In the presence of this complex, Rad17 and 9-1-1 are subsequently recruited. The ATR-ATRIP pathway is finally activated with the aid of DNA topoisomerase II binding protein 1 (TOPBP1). HR is mediated by proteins of BRCA1, PALB2, and BRCA2 in association with effector proteins Rad51 and Rad52 (**Figure 2**).

As described earlier, TMZ induced O6-MeG mismatch lead to the DSB by the futile cycle (Roos and Kaina, 2012). In addition, although TMZ does not involve DSB formation directly, it has been reported that TMZ can provoke HR with a potency more than 10-fold that of ionizing radiation, which is a known DSB inducer (Helleday, 2010). These results indicate that signaling molecules implicated in the DSB pathway play a role in mediating TMZ sensitivity. Indeed, NBS1 (a member of the MRN complex), Rad51, and BRCA2, all signaling proteins implicated in the DSB pathway, have been reported to be modulators of TMZ sensitivity in an *in vitro* tumor model (Eich et al., 2010; Quiros et al., 2011; Short et al., 2011). Moreover, several studies have shown that ATM, ATR, and MRN proteins can contribute the TMZ-induced G2 arrest and cytotoxicity in a MMR-dependent manner, suggesting that these molecules may be potential targets to overcome TMZ resistance (Caporali et al., 2004; Stojic et al., 2004b; Mirzoeva et al., 2006; Schroering et al., 2009).

### FANCONI ANEMIA PATHWAY

Fanconi anemia is genomic instability syndrome characterized by developmental abnormalities in major organ systems, bone marrow failure, and a high predisposition to cancer (D’Andrea and Grompe, 2003). FA is currently known to be caused by the mutation of at least one of the 15 FA family genes (Kim and D’Andrea, 2012). The FA pathway is believed to be implicated in the repair of DNA interstrand cross-linking by cisplatin and topoisomerase inhibitors, and to mediate resistance to this type of drug. However, recent evidence demonstrated that the FA pathway may be implicated in the repair process of alkylation damage by alkylating agents and in the determination of the therapeutic sensitivity to alkylating agents (Chen et al., 2007; Kondo et al., 2011).

In the FA pathway, FANCM recognize DNA ICL and initiate the repair pathway by recruiting eight FA core complexes (FANCA/B/C/E/F/G/L/M) to the damaged site. This FA core protein complex monoubiquitinates FANCD2 and FANCI, which interact with DNA repair proteins such as BRCA1, BRCA2 (FANCD1), PALB2 (FANCN), and FANCJ (BACH1/BRIP1), facilitating the repair process (Kee and D’Andrea, 2010; **Figure 2**). Of all these steps, monoubiquitination of FANCD2 and BRCA2 (FANCD1) is the critical step in the FA repair pathway. A recent study by Chen et al. (2007) demonstrated that inhibition of FANCD2 monoubiquitination sensitizes the glioma cell line to the cytotoxic effects of TMZ, and suggested that the FA pathway may play some role in TMZ resistance. Furthermore, other recent studies showed that BRCA2 (FANCD1) plays a predominant role in the repair of DNA damage induced by TMZ, and inhibiting BRCA2 sensitize the glioma cell lines to TMZ treatment, suggesting that BRCA2 is also a molecular target for overcoming TMZ resistance (Kondo et al., 2011; Quiros et al., 2011). Given that BRCA2 (FANCD1) functions in mediating HR, this result, as well as recent studies demonstrating that the FA pathway also functions in the repair of DSB, suggests that more detailed investigation into the FA pathway may reveal novel mechanistic findings related to the regulation of the cellular processing after TMZ-induced cytotoxicity.

## CONCLUDING REMARKS

Although the investigation of DNA repair pathways in the tolerance of TMZ chemotherapy has been focused on the MGMT and MMR pathways, recent research has demonstrated that the BER pathway also plays a significant role in determining the sensitivity to TMZ. The possible roles of the SSB and DSB repair pathways in mediating therapeutic resistance to TMZ have also begun to emerge. Given that each DNA repair pathway does not function independently of the others, but shows redundancy and crosstalk, it is possible that a more significant role for DNA strand repair pathways, such as PARP and HR, will be revealed in relation to TMZ treatment. The response to DNA damage is dependent on the extent of the DNA damage inflicted. Thus, detailed analysis of complicated repair pathways can aid in identifying new mechanisms of chemoresistance. Moreover, establishing useful molecular markers to assess the status of DNA repair in clinical tissue is mandatory for future studies to develop an effective treatment strategy targeting repair pathways (Jalal et al., 2011). Finally, chemoresistance is defined not only by the tumor itself, but also by the tumor microenvironment. The brain tumor stem cell population is another critical biological factor in chemoresistance because the DNA repair pathway is activated in stem cell populations. Further investigation should be performed taking these factors into consideration.

## Conflict of Interest Statement

The authors declare that the research was conducted in the absence of any commercial or financial relationships that could be construed as a potential conflict of interest.
